# Mixed‐Valence Compounds as Polarizing Agents for Overhauser Dynamic Nuclear Polarization in Solids**

**DOI:** 10.1002/anie.202103215

**Published:** 2021-06-07

**Authors:** Andrei Gurinov, Benedikt Sieland, Andrey Kuzhelev, Hossam Elgabarty, Thomas D. Kühne, Thomas Prisner, Jan Paradies, Marc Baldus, Konstantin L. Ivanov, Svetlana Pylaeva

**Affiliations:** ^1^ NMR Spectroscopy group Bijvoet Center for Biomolecular Research Utrecht University Padualaan 8 3584CH Utrecht The Netherlands; ^2^ Department of Chemistry Paderborn University Warburger Strasse 100 Paderborn 33098 Germany; ^3^ Goethe University Frankfurt am Main Institute of Physical and Theoretical Chemistry Center for Biomolecular Magnetic Resonance Max von Laue Strasse 7 60438 Frankfurt am Main Germany; ^4^ Dynamics of Condensed Matter and Center for Sustainable Systems Design Chair of Theoretical Chemistry University of Paderborn Warburger Strasse 100 33098 Paderborn Germany; ^5^ International Tomography Center Siberian Branch of the Russian Academy of Sciences Novosibirsk 630090 Russia; ^6^ Novosibirsk State University Novosibirsk 630090 Russia

**Keywords:** ab initio calculations, DNP in solids, mixed valence compounds, molecular dynamics, Overhauser effect

## Abstract

Herein, we investigate a novel set of polarizing agents—mixed‐valence compounds—by theoretical and experimental methods and demonstrate their performance in high‐field dynamic nuclear polarization (DNP) NMR experiments in the solid state. Mixed‐valence compounds constitute a group of molecules in which molecular mobility persists even in solids. Consequently, such polarizing agents can be used to perform Overhauser‐DNP experiments in the solid state, with favorable conditions for dynamic nuclear polarization formation at ultra‐high magnetic fields.

## Graphical abstract

Mixed‐valence compounds were investigated as novel polarizing agents for DNP NMR. These compounds constitute a group of molecules in which molecular mobility persists even in solids. Consequently, they can be used to perform Overhauser‐DNP NMR experiments in solid state, and they specifically show favorable properties for applications at ultra‐high magnetic fields (DNP=dynamic nuclear polarization, NMR=nuclear magnetic resonance).

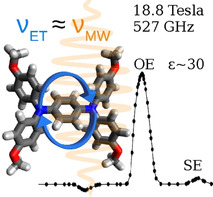

Dynamic nuclear polarization (DNP)[^1^, ^2^] has become a widely used method for signal enhancement in various nuclear magnetic resonance (NMR) experiments. DNP NMR has allowed applications that were not deemed feasible before: from proteins in cells,^[3]^ to atomistic studies of mesoporous materials,[^4^, ^5^] and to clinical applications of dissolution DNP.^[6]^ The idea of DNP is to transfer equilibrium polarization of electron spins to nuclear spins by pumping electron paramagnetic resonance (EPR) transitions of stable paramagnetic compounds added to the sample, i.e., “polarizing agents”. Ideally, the NMR signal enhancements are reaching a value equivalent to the ratio *γ*_e_ */γ*_N_ (with *γ*_e_ and *γ*_N_ being the electron and nuclear gyromagnetic ratios), which is equal to 660 for protons, i.e., for nuclei with the highest *γ*‐ratio. However, achieving the maximal theoretically allowed enhancement (or even approaching it) is still a big challenge, notably, in high magnetic fields where the polarization transfer efficiency is expected to decrease.

Historically, the first DNP mechanism to be discovered was the Overhauser mechanism,^[7]^ relying on electron–nuclear cross‐relaxation and thus requiring fluctuations of the electron–nuclear hyperfine coupling (HFC). In insulating solids, the Overhauser mechanism was deemed to be inefficient. For this reason, solid‐state DNP has relied on other mechanisms, known as solid effect,[^8^, ^9^] cross effect[^8^, ^10^, ^11^] and thermal mixing.^[12]^ However, quite surprisingly, in some cases the Overhauser effect is operative in insulating solids,^[13]^ moreover, the enhancement scales favorably with the magnetic field (increasing upon the field increase from 9.4 to 18.8 Tesla).^[14]^ It is worth noting that in the case of Overhauser DNP, microwave pumping is performed on allowed NMR transitions, which are easier to saturate, providing a possible solution to the problem of limited microwave power available at high frequencies. Since Overhauser DNP in solids is an efficient mechanism at high magnetic fields (used to improve the NMR resolution) investigation of this phenomenon and further optimization of the enhancement are of great interest. So far, Overhauser DNP in insulating solids has been reported for a single specific polarizing agent—the BDPA radical.[^13^, ^14^] Ab initio electronic structure calculations have shown that this radical is a mixed‐valence compound^[15]^ in which the electron spin density is spontaneously hopping between the two sites, giving rise to fluctuations of the HFC and, consequently, to cross‐relaxation.[^16^, ^17^] According to molecular dynamics simulations the spectral density of the fluctuations is peaking at frequencies around 100–700 GHz, providing favorable conditions for electron–nuclear cross‐relaxation at high fields and thus to DNP enhancements.^[17]^ Interestingly, a similar mixed‐valence behavior was reported even before the BDPA for a flavin derivative.^[18]^ Presently, experimental data on Overhauser DNP in insulating solids are limited, as the effect has been reported for a single polarizing agent. The goal of this work is thus (i) to verify the theoretical prediction that mixed‐valence compounds are suitable polarizing agents for Overhauser DNP and potentially (ii) improving the enhancement provided by Overhauser DNP in solids at high magnetic fields. To this end, we here study a new set of mixed‐valence radicals, namely *N* ^1^,*N* ^1^,*N* ^4^,*N* ^4^‐tetrakis(4‐methoxyphenyl)benzene‐1,4‐diamine (1‐4‐amine) radical, and *N* ^1^,*N* ^1^,*N* ^3^,*N* ^3^‐tetrakis(4‐methoxyphenyl)benzene‐1,3‐diamine (1‐3‐amine) radical shown in Figure 1 and compare their performance in DNP experiments to those of the 1,3‐bis(diphenylene)‐2‐phenylallyl (BDPA) radical. Based on our previous work, these radicals were chosen from a theoretical screening of a larger number of potential mixed‐valence candidates using electronic structure calculations. The most promising candidates were synthesized, and we here present the data of an EPR study of these new radicals and also the results of DNP experiments performed at the magnetic field of 18.8 Tesla. Our results indeed show that mixed‐valence compounds are suitable polarizing agents for DNP and support the idea that Overhauser DNP in solids is due to the transitions between “alternative” valence structures of such compounds, which give rise to the required fluctuations of HFCs.

**Figure 1:**
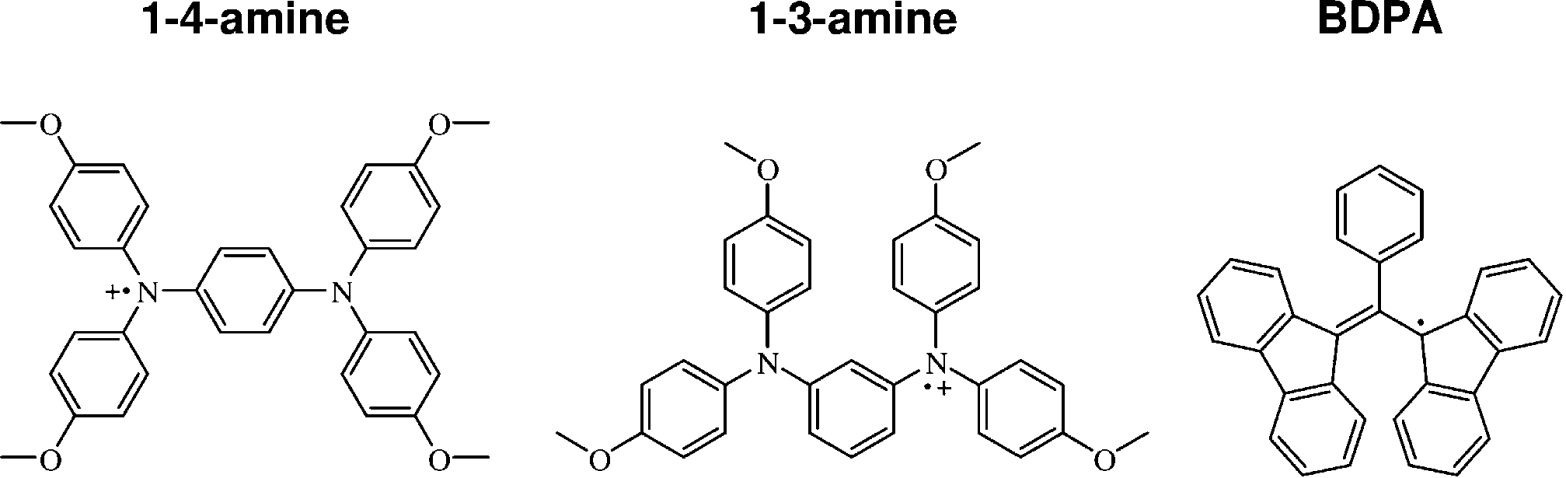
Set of the radicals investigated: *N* ^1^,*N* ^1^,*N* ^4^,*N* ^4^‐tetrakis(4‐methoxyphenyl)benzene‐1,4‐diamine (1‐4‐amine), *N* ^1^,*N* ^1^,*N* ^3^,*N* ^3^‐tetrakis(4‐methoxyphenyl)benzene‐1,3‐diamine (1‐3‐amine), and 1,3‐bis(diphenylene)‐2‐phenylallyl (BDPA) radical.

Compounds like BDPA are known as mixed‐valence compounds. Such systems are also called (pseudo) Jahn–Teller systems,[^15^, ^19^] where electronic and vibrational degrees of freedom are coupled. According to a classification scheme by Robin and Day,^[20]^ BDPA belongs specifically to the class II mixed‐valence compounds.^[16]^ Such compounds have a localized electronic state with a barrier in the center. Accordingly, the two valence states exhibit a coupling interaction of intermediate strength. The interaction is sufficiently weak to prevent a collapse into one symmetric state but strong enough to reduce the height of the energy barrier in the middle. In general, the interconversion of two structures occurs upon excitation: thermally, when higher vibrational states are populated, or via tunneling through the barrier.[^21^, ^22^] It is worth mentioning that changes of HFC pattern with temperature are often used to estimate the electron transfer rate in mixed‐valence compounds.[^15^, ^23^] Recently, some of us have confirmed that BDPA belongs to class II mixed‐valence compounds using high level electronic structure methods.^[16]^ Novel radicals have been chosen based on intensive literature search with a few considerations in mind, i.e., fast electron transfer rate and narrow EPR line.[^15^, ^24^, ^25^] Both 1‐3‐amine and 1‐4‐amine have been reported to have a mixed‐valence character close to the class II/III border.[^15^, ^23^, ^26^] Calculations of the *g*‐tensor revealed that both radicals have relatively narrow EPR lines at high fields (*g*_aniso_(1‐3‐amine)=0.0006, *g*_aniso_(1‐4‐amine)=0.0007, *g*_aniso_(BDPA)=0.0003, *g*_aniso_=(*g*_zz_−*g*_xx_)/*g*_iso_). In 1‐3‐amine spin density is mostly localized on one side of the molecule, which is indicated by values of the isotropic hyperfine coupling constants (see Figure 2, a full list of HFC constants is provided in the Supporting Information). DFT calculations of 1‐4‐amine in vacuum yielded a class III structure where spin density is delocalized over the entire molecule.^[27]^ However, preliminary CASSCF calculations point to a class II structure. Furthermore, solvent and counter‐ion effects are known to influence the class of mixed‐valence compounds: polar solvents as well as more compact counter‐ions tend to stabilize localized class II structures.

**Figure 2:**
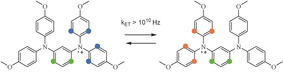
Changes in hyperfine coupling pattern due to electron transfer in 1‐3‐amine (BMK/EPR‐III calculation). Rate of the electron transfer was estimated by Uebe et al.^[23]^

EPR spectra of the compounds under study obtained at 6.4 Tesla are shown in Figure 3, along with the spectrum of BDPA shown for comparison. Both 1‐3‐amine and 1‐4‐amine exhibit a narrow EPR line corresponding to *g=*2.00359, which is inhomogeneously broadened due to *g*‐tensor anisotropy. Compared to BDPA, the resonance is found at a lower field, and the EPR line is almost two times broader. However, the EPR linewidth (when measured in frequency units) is still smaller than the NMR frequency *ω*_N_ at the same field. Consequently, in DNP experiments one should expect clearly resolved contributions from the solid effect and Overhauser effect, as previously observed for BDPA.^[13]^

**Figure 3:**
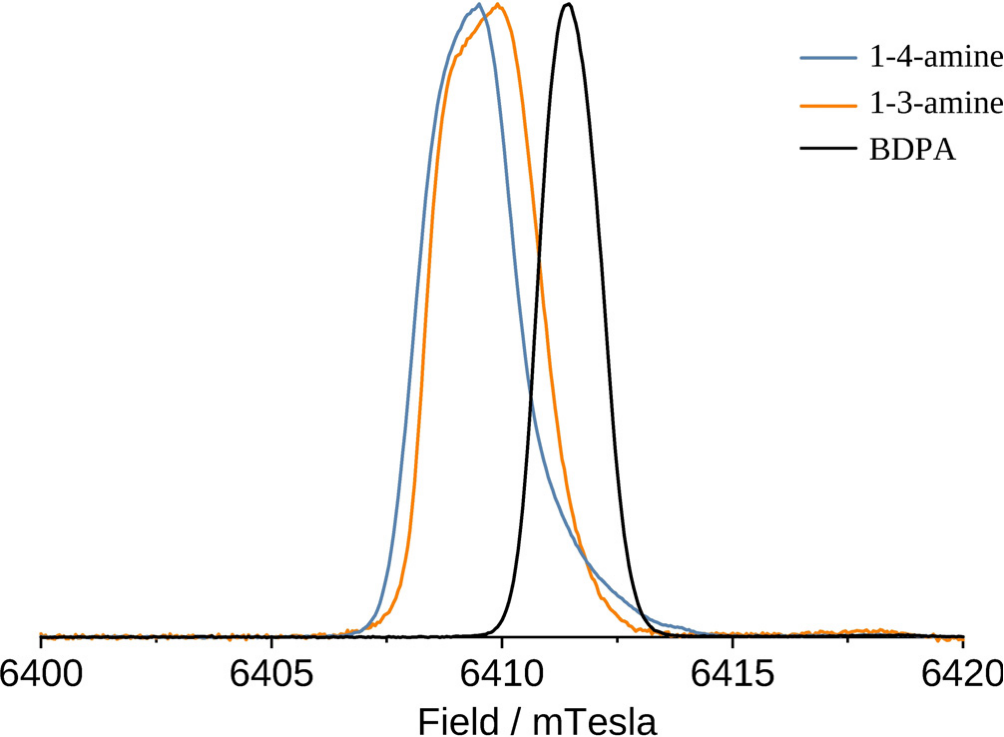
EPR spectra of the solutions of radicals in TCE at 50 K, acquired on a 180 GHz EPR spectrometer.

Figure 4 shows the DNP enhancement measured at a constant microwave frequency of 527 GHz as a function of the external magnetic field, which was incremented in small steps between subsequent DNP measurements. In accordance with our expectations, individual components are clearly resolved in each spectrum, with the outer components (negative component at lower field and positive component at higher field) corresponding to the solid effect and the central component corresponding to Overhauser DNP, in qualitative agreement with studies on BDPA reported before.^[13]^ However, the newly proposed radicals show a better performance not only in terms of the maximum enhancement (found for the central component corresponding to Overhauser DNP) but also in terms of the ratio of the enhancement determined for the central component and outer components (which stands for the relative efficiency of Overhauser DNP and solid‐effect DNP). The maximal enhancement factor found here reaches approximately 30 for 1‐4‐amine and 20 for 1‐3‐amine, whereas we found for BDPA a maximum enhancement of 8 under the same conditions.

**Figure 4:**
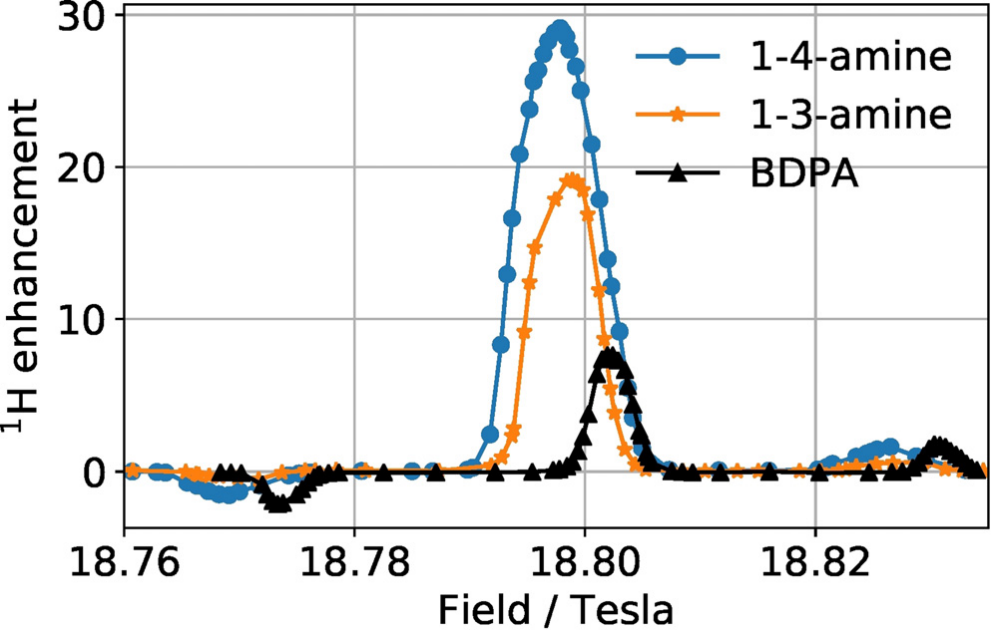
Field profiles for the set of radicals measured at 100 K in TCE (90D:10H) matrix under 8 kHz MAS. Sample preparation details are given in the SI.

To gain additional insight into the DNP process we have also measured the dependence of the enhancement on the pumping power for the central component and one of the outer components. Such dependencies are expected to be different^[13]^ because in the former case pumping is performed on the “allowed” EPR transition, whereas in the latter case “forbidden” EPR transitions are irradiated. Hence, different transitions are expected to be saturated at different microwave power. As shown in Figure 5 this is indeed the case for both radicals under study. Notably, for the central transition the enhancement approaches its maximal value at the power of 3.5 Watt, whereas for the forbidden transitions the enhancement keeps increasing even at the highest available power. The enhancement coming from the Overhauser effect thus scales favorably with microwave power, in contrast to solid‐effect DNP. Lastly, we also investigated the MAS dependence (Figure 6) for the new radicals, which showed a similar behavior to previous OE DNP studies.^[28]^

**Figure 5:**
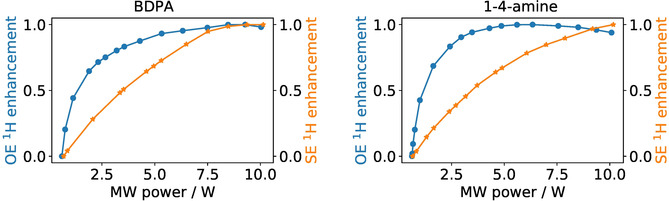
Normalized ^1^H DNP enhancement as a function of MW power for different components of the field profile: for BDPA (left) and 1‐4‐amine (right). Data for 1‐3‐amine are shown in Figure S2 in the SI.

**Figure 6:**
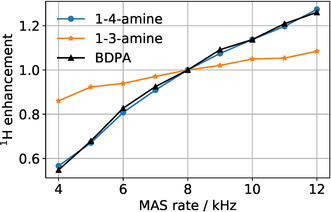
Normalized enhancement as a function of MAS frequency for the studied radicals.

In summary, we have experimentally observed novel mixed‐valence radicals inducing hyperpolarization based on the Overhauser mechanism in insulating solids. Our findings are based on a set of complementary methods: computer simulations, high field EPR and DNP measurements. We plan to continue our investigation of mixed‐valence polarizing agents with special focus on their stability under physiological (i.e., aqueous solution, higher temperature) conditions both experimentally and theoretically.

## Experimental Section

Computational details. DFT calculations were performed using Gaussian16.^[29]^ Geometry optimization was performed at the BMK/TZVPP level,[^30^, ^31^] followed by calculations of magnetic properties at the BMK/EPR‐III level;^[32]^ ultrafine convergence and integral treatment were employed in all calculations. CASSCF calculations were done in ORCA at the CASSCF(3,3)/def2‐TZVP level of theory.[^31^, ^33^]

Experimental details. Synthesis and sample preparation details are given in the Supporting Information. Echo‐detected EPR spectra were obtained using a home‐built Gband EPR spectrometer (180 GHz, 6.4 T).^[34]^ EPR spectra were recorded in a 1,1,2,2‐tetrachloroethane (TCE) matrix with a radical concentration of 0.1–0.5 mm. G‐band echo‐detected EPR spectra were recorded at 50 K using a pulse length of 44 ns and 70 ns for π/2 and π pulses, respectively; the inter‐pulse delay was 200 ns. To determine the values of the *g*‐factor of nitroxides, we placed a ^55^Mn^2+^ standard sample (*g*(Mn^2+^)=2.00101) in the resonator together with the studied sample. The *g*‐tensor parameters of all radicals under study were obtained from simulation of the G‐band EPR spectra with the EasySpin program^[35]^ using function pepper in the corresponding solid‐state regime.

DNP experiments were performed on an 800 MHz/527 GHz NMR/DNP spectrometer (Bruker BioSpin) equipped with a sweep coil that allowed to vary the *B*_0_ magnetic field in the range of ±45 mT. The MAS frequency was set to 8 kHz unless stated otherwise. The DNP enhancement was obtained by comparing the ^1^H signals of TCE with and without MW irradiation using a rotor‐synchronized Hahn echo pulse sequence after a series of saturation pulses. The DNP build‐up times *T*_B_ as well as the spin–lattice relaxation times *T*_1_ for the radicals under investigation were measured using the same pulse sequence by altering recovery times. While the *T*_1_ times were virtually the same for all radicals 35 s, the *T*_B_ were 36 and 44 s for 1‐3‐amine and 1‐4‐amine, respectively.

## Conflict of interest

The authors declare no conflict of interest.

## Supporting information

As a service to our authors and readers, this journal provides supporting information supplied by the authors. Such materials are peer reviewed and may be re‐organized for online delivery, but are not copy‐edited or typeset. Technical support issues arising from supporting information (other than missing files) should be addressed to the authors.

Supplementary

## References

[1] T. Maly, G. T. Debelouchina, V. S. Bajaj, K.-N. Hu, C. G. Joo, Mak-M. L. Jurkauskas, J. R. Sirigiri, P. C. A. van der Wel, J. Herzfeld, R. J. Temkin, R. G. Griffin, J. Chem. Phys. 2008, 128, 052211. 18266416 10.1063/1.2833582 PMC2770872

[2] B. Corzilius, Annu. Rev. Phys. Chem. 2020, 71, 143–170. 32074473 10.1146/annurev-physchem-071119-040222

[3] S. Narasimhan, S. Scherpe, A. Lucini Paioni, J. van der Zwan, G. E. Folkers, H. Ovaa, M. Baldus, Angew. Chem. Int. Ed. 2019, 58, 12969–12973; 10.1002/anie.201903246 PMC6772113 31233270

[4] A. J. Rossini, A. Zagdoun, M. Lelli, A. Lesage, C. Copéret, L. Emsley, Acc. Chem. Res. 2013, 46, 1942–1951. 23517009 10.1021/ar300322x

[5] T. Kobayashi, F. A. Perras, I. I. Slowing, A. D. Sadow, M. Pruski, ACS Catal. 2015, 5, 7055–7062.

[6] J. H. Ardenkjaer-Larsen, J. Magn. Reson. 2016, 264, 3–12. 26920825 10.1016/j.jmr.2016.01.015

[7] A. W. Overhauser, Phys. Rev. 1953, 92, 411–415.

[8] C. D. Jeffries, Phys. Rev. 1957, 106, 164–165.

[9] Y. Hovav, A. Feintuch, S. Vega, J. Magn. Reson. 2010, 207, 176–189. 21084205 10.1016/j.jmr.2010.10.016

[10] C. F. Hwang, D. A. Hill, Phys. Rev. Lett. 1967, 19, 1011–1014.

[11] Y. Hovav, A. Feintuch, S. Vega, J. Magn. Reson. 2012, 214, 29–41. 22119645 10.1016/j.jmr.2011.09.047

[12] A. Abragam, M. Goldman, Rep. Prog. Phys. 1978, 41, 395–467.

[13] T. V. Can, M. A. Caporini, F. Mentink-Vigier, B. Corzilius, J. J. Walish, M. Rosay, W. E. Maas, M. Baldus, S. Vega, T. M. Swager, R. G. Griffin, J. Chem. Phys. 2014, 141, 064202. 25134564 10.1063/1.4891866 PMC4137812

[14] M. Lelli, S. R. Chaudhari, D. Gajan, G. Casano, A. J. Rossini, O. Ouari, P. Tordo, A. Lesage, L. Emsley, J. Am. Chem. Soc. 2015, 137, 14558–14561. 26555676 10.1021/jacs.5b08423 PMC4671100

[15] A. Heckmann, C. Lambert, Angew. Chem. Int. Ed. 2012, 51, 326–392; 10.1002/anie.201100944 22095940

[16] S. Pylaeva, P. Marx, G. Singh, T. D. Kühne, M. Roemelt, H. Elgabarty, J. Phys. Chem. A 2021, 125, 867–874. 33464904 10.1021/acs.jpca.0c11296

[17] S. Pylaeva, K. L. Ivanov, M. Baldus, D. Sebastiani, H. Elgabarty, J. Phys. Chem. Lett. 2017, 8, 2137–2142. 28445055 10.1021/acs.jpclett.7b00561

[18] T. Maly, D. Cui, R. G. Griffin, A.-F. Miller, J. Phys. Chem. B 2012, 116, 7055–7065. 22472179 10.1021/jp300539j PMC6010065

[19] I. B. Bersuker, Chem. Rev. 2013, 113, 1351–1390. 23301718 10.1021/cr300279n

[20] M. B. Robin, P. Day, Adv. Inorg. Chem. Radiochem., Elsevier, Amsterdam, 1968, pp. 247–422.

[21] A. H. Schroeder, S. Mazur, J. Am. Chem. Soc. 1978, 100, 7339–7346.

[22] P. Fuerderer, F. Gerson, J. Heinzer, S. Mazur, H. Ohya-Nishiguchi, A. Schroeder, J. Am. Chem. Soc. 1979, 101, 2275–2281.

[23] M. Uebe, A. Ito, Chem. Asian J. 2019, 14, 1692–1696. 30698917 10.1002/asia.201900036

[24] J. Hankache, O. S. Wenger, Chem. Rev. 2011, 111, 5138–5178. 21574545 10.1021/cr100441k

[25] M. Parthey, M. Kaupp, Chem. Soc. Rev. 2014, 43, 5067–5088. 24781049 10.1039/c3cs60481k

[26] C. Lambert, S. Amthor, J. Schelter, J. Phys. Chem. A 2004, 108, 6474–6486.

[27] M. Renz, K. Theilacker, C. Lambert, M. Kaupp, J. Am. Chem. Soc. 2009, 131, 16292–16302. 19831383 10.1021/ja9070859

[28] S. R. Chaudhari, D. Wisser, A. C. Pinon, P. Berruyer, D. Gajan, P. Tordo, O. Ouari, C. Reiter, F. Engelke, C. Copéret, M. Lelli, A. Lesage, L. Emsley, J. Am. Chem. Soc. 2017, 139, 10609–10612. 28692804 10.1021/jacs.7b05194 PMC5719465

[29] M. J. Frisch, et al., Gaussian 16 Revision, C.01. 2016, Gaussian Inc. Wallingford, CT.

[30] A. D. Boese, J. M. L. Martin, J. Chem. Phys. 2004, 121, 3405–3416. 15303903 10.1063/1.1774975

[31] F. Weigend, Phys. Chem. Chem. Phys. 2006, 8, 1057–1065. 16633586 10.1039/b515623h

[32] D. P. Chong, Recent Advances in Density Functional Methods, World Scientific, Singapore, 1995.

[33] F. Neese, WIREs Comput. Mol. Sci. 2018, 8, e1327.

[34] M. Rohrer, O. Brügmann, B. Kinzer, T. Prisner, Appl. Magn. Reson. 2001, 21, 257–274.

[35] S. Stoll, A. Schweiger, J. Magn. Reson. 2006, 178, 42–55. 16188474 10.1016/j.jmr.2005.08.013

