# Supplementary material for: Mixed‐Valence Compounds as Polarizing Agents for Overhauser Dynamic Nuclear Polarization in Solids
Source: Angew Chem Int Ed Engl. 2021 Jun 7;60(28):15371–5. doi: 10.1002/anie.202103215 (PMC8361920; doi:10.1002/anie.202103215)
Supplement: Supplementary file 1 — Supplementary [file ANIE-60-15371-s001.pdf]

Angewandte Chemie
Eine Zeitschrift der Gesellschaft Deutscher Chemiker

Supporting Information

Mixed-Valence Compounds as Polarizing Agents for Overhauser Dynamic Nuclear Polarization in Solids**

*Andrei Gurinov, Benedikt Sieland, Andrey Kuzhelev, Hossam Elgabarty, Thomas D. Kühne, Thomas Prisner, Jan Paradies, Marc Baldus, Konstantin L. Ivanov†, and Svetlana Pylaeva**

anie_202103215_sm_miscellaneous_information.pdf

1. Synthesis

1.1. Synthesis of N^1,N^1,N^4,N^4-tetrakis(4-methoxyphenyl)benzene-1,4-diamine[1]

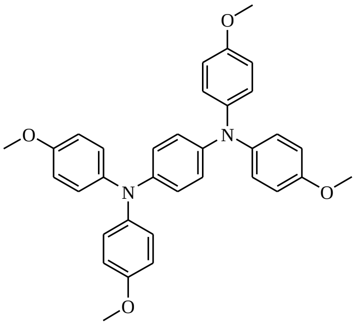

The synthesis was performed analogous to the synthesis of triarylated amines. A 10 mL crimp vial with magnetic stir bar was charged with 4-iodoanisol (257.4 mg, 1.10 mmol, 4.4 eq.), tris(dibenzylidene-acetone)dipalladium(0) (8.3 mg, 0.01 mmol, 0.04 eq.), XPhos (26.0 mg, 0.06 mmol, 0.22 eq.), sodium *tert*-butoxide (147.7 mg, 1.54 mmol, 6.16 eq.) and 1,4-diamino benzene (27.0 mg, 0.25 mmol, 1.0 eq.) and 1,4-dioxane (1.65 mL). The mixture was stirred for 5 h at 110°C, then cooled to RT and quenched with EtOAc (4 mL). After filtration over Celite, the solvent was removed under reduced pressure. Column chromatography (silica, gradient CH/EE 10:1 to 5:1, R_f(5:1)=0.49) and precipitation three times from MeOH (100 mL) gave a nearly colourless, amorphous solid (722.6 mg, 68 %).

^{1}H-NMR (500 MHz, C_6D_6, 303 K, δ [ppm]): 7.15-7.11 (m, 8H, CH_{Ar}), 7.07 (br., 4H, CH_{Ar}), 6.76-6.69 (m, 8H, CH_{Ar}), 3.31 (s, 12H, CH_3).

^{13}C-NMR (176 MHz, C_6D_6, 303 K, δ [ppm]): 156.4 (4C, C_q), 143.9 (2C, C_q), 142.6 (4C, C_q), 126.4 (8C, CH_{Ar}), 124.2 (4C, CH_{Ar}), 115.5 (8C, CH_{Ar}), 55.4 (4C, CH_3).

^{15}N-NMR (71 MHz, C_6D_6; 303 K, δ [ppm]): 91.4.

Mass (ESI; [m/z]): calculated [M^+] 532.2362, found [M^+] 532.2368.

1.2. Synthesis of N^1,N^1,N^3,N^3-tetrakis(4-methoxyphenyl)benzene-1,3-diamine[1]

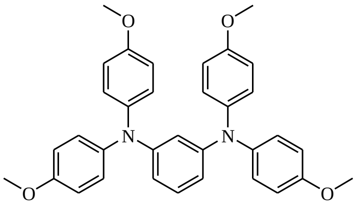

The synthesis was performed analogous to the synthesis of triarylated amines. A 10 mL crimp vial with magnetic stir bar was charged with 4-iodoanisol (257.4 mg, 1.10 mmol, 4.4 eq.), tris(dibenzylidene-acetone)dipalladium(0) (8.3 mg, 0.01 mmol, 0.04 eq.), XPhos (26.0 mg, 0.06 mmol, 0.22 eq.), sodium *tert*-butoxide (147.7 mg, 1.54 mmol, 6.16 eq.), 1,3-diamino benzene (27.0 mg, 0.25 mmol, 1,0 eq.) and 1,4-dioxane (1.65 mL). The mixture was stirred for 5 h at 110°C, then cooled to RT and quenched with EtOAc (4 mL). After filtration over Celite the solvent was removed under reduced pressure. Column chromatography (silica, gradient CH/EE 10:1 to 5:1, R_f(10:1)=0.19) and precipitation three times from MeOH (100 mL) at -20 °C gave a nearly colourless, amorphous solid (391,0 mg; 37 %).

^{1}H-NMR (300 MHz, C_6D_6, 303 K, δ [ppm]): 7.13-7.07 (m, 8H, CH_{Ar}), 7.04 (t, $^4J_{HH}$=2.2 Hz, 4H, CH_{Ar}), 7.02 (t, $^3J_{HH}$=8.1 Hz, 1H, CH_{Ar}), 6.73 (dd, $^3J_{HH}$=8.1 Hz, $^4J_{HH}$=2.3 Hz, 2H, CH_{Ar}), 6.70-6.64 (m, 8H, CH_{Ar}), 3.29 (s, 12H, CH_3).

^{13}C-NMR (176 MHz, C_6D_6, 303 K, δ [ppm]): 156.6 (4C, C_q), 150.5 (2C, C_q), 142.0 (4C, C_q), 130.3 (1C, CH_{Ar}), 127.0 (8C, CH_{Ar}), 115.4 (9C, CH_{Ar}), 114.8 (2C, CH_{Ar}), 55.3 (4C, CH_3).

^{15}N-NMR (71 MHz, C_6D_6, 303 K, δ [ppm]): 94.5.

Mass (ESI, [m/z]): calculated [M^+] 532.2362, found [M^+] 532.2363.

2. Sample preparation

For DNP measurements, the corresponding closed shell molecule (15 µmol, 1 eq.) was dissolved in 1,1,2,2-tetrachloroethane (1.0 mL, 90D:10H) and the solution was added to the oxidizing agent (15 µmol, 2.6 mg, 1 eq.). The mixture was vigorously shaken, and a sample was immediately taken for measurement. This yielded a 15 mM concentration of the radical in the sample.

Substance	CAS-number	molecular weight [g/mol]	amount of substance [mmol]	mass [mg]	volume [mL]
N1,N1,N3,N3-Tetrakis(4-methoxyphenyl)benzene-1,3-diamine	No CAS-No.	532.64	0.015	8.0	
N1,N1,N4,N4-Tetrakis(4-methoxyphenyl)benzene-1,4-diamine	No CAS-No.	532.64	0.015	8.0	
Nitrosyl hexafluorophosphate	16921-91-8	174.97	0.015	2.6	
1,1,2,2-Tetrachlorethane-d2	33685-54-0				0.9
1,1,2,2-Tetrachlorethane					0.1
α,γ-Bisdiphenylene-β-phenylallyl	35585-94-5	495.63	0.02	7.4	

For EPR measurements, the corresponding closed shell molecule (15 µmol, 1 eq.) was dissolved in 1,1,2,2-tetrachloroethane (1.0 mL); and the solution was added to the oxidizing agent (15 µmol, 2.6 mg, 1 eq.). The mixture was vigorously shaken. 0.01 - 0.03 mL of the mixture was taken out of the solution and dissolved in 0.9 ml of 1,1,2,2-tetrachloroethane. A sample was immediately taken for measurement. This yielded a 0.1 - 0.5 mM concentration of the radical in the sample.

Substance	CAS-number	molecular weight [g/mol]	amount of substance [mmol]	mass [mg]	volume [mL]
N1,N1,N3,N3-Tetrakis(4-methoxyphenyl)benzene-1,3-diamine	No CAS-No.	532.64	0.015	8.0	
N1,N1,N4,N4-Tetrakis(4-methoxyphenyl)benzene-1,4-diamine	No CAS-No.	532.64	0.015	8.0	
Nitrosyl hexafluorophosphate	16921-91-8	174.97	0.015	2.6	
1,1,2,2-Tetrachlorethane	33685-54-0				1.9
α,γ-Bisdiphenylene-β-phenylallyl	35585-94-5	495.63	0.02	7.4	

3. Simulations results

DFT Results: BMK/EPR-III calculation of hyperfine coupling constants

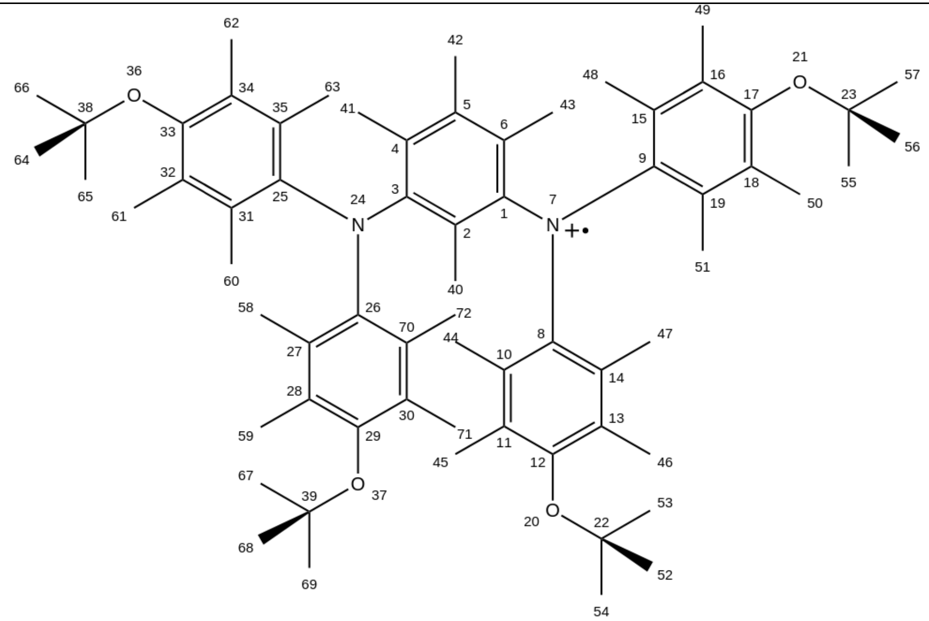

Atom	MHz	Atom	MHz	Atom	MHz	Atom	MHz
1 C(13)	-26.38176	19 C(13)	15.60523	37 O(17)	-0.19667	55 H(1)	-0.18926
2 C(13)	8.04364	20 O(17)	-9.12289	38 C(13)	-0.06753	56 H(1)	3.66216
3 C(13)	-12.12234	21 O(17)	-7.18166	39 C(13)	-0.04496	57 H(1)	4.24656
4 C(13)	25.80272	22 C(13)	-2.05904	40 H(1)	-2.66010	58 H(1)	-0.17507
5 C(13)	-21.27062	23 C(13)	-1.60951	41 H(1)	-12.50268	59 H(1)	0.18644
6 C(13)	26.70952	24 N(14)	1.30940	42 H(1)	4.33924	60 H(1)	-0.05633
7 N(14)	26.20533	25 C(13)	-0.56482	43 H(1)	-11.46542	61 H(1)	-0.10051
8 C(13)	-20.99350	26 C(13)	-1.09473	44 H(1)	-5.49117	62 H(1)	0.10372
9 C(13)	-22.43819	27 C(13)	0.74783	45 H(1)	1.89767	63 H(1)	0.47310
10 C(13)	11.75601	28 C(13)	-0.33563	46 H(1)	2.30399	64 H(1)	-0.00279
11 C(13)	-10.72689	29 C(13)	0.27825	47 H(1)	-7.27108	65 H(1)	0.20873
12 C(13)	15.00310	30 C(13)	-0.00045	48 H(1)	-4.91768	66 H(1)	0.16597
13 C(13)	-13.48376	31 C(13)	-0.02234	49 H(1)	2.58171	67 H(1)	-0.00597
14 C(13)	15.25525	32 C(13)	0.32483	50 H(1)	1.74727	68 H(1)	0.11156
15 C(13)	12.22226	33 C(13)	0.18880	51 H(1)	-6.80798	69 H(1)	0.10223
16 C(13)	-10.71614	34 C(13)	-0.49916	52 H(1)	-0.20384	70 C(13)	1.25622
17 C(13)	12.82228	35 C(13)	-0.16172	53 H(1)	4.75794	71 H(1)	0.01550
18 C(13)	-10.33527	36 O(17)	-0.24527	54 H(1)	5.67063	72 H(1)	-0.18196

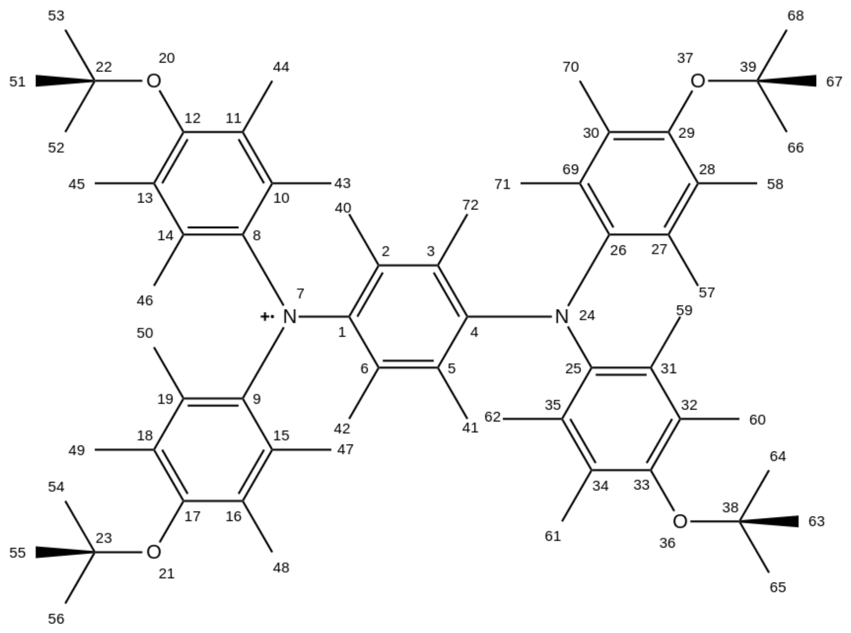

Atom	MHz	Atom	MHz	Atom	MHz	Atom	MHz
1 C(13)	-2.22777	19 C(13)	10.11654	37 O(17)	-2.72128	55 H(1)	1.30580
2 C(13)	0.80459	20 O(17)	-2.72136	38 C(13)	-0.61766	56 H(1)	1.71791
3 C(13)	0.80461	21 O(17)	-2.72137	39 C(13)	-0.61766	57 H(1)	-2.78046
4 C(13)	-2.22772	22 C(13)	-0.61769	40 H(1)	-2.13757	58 H(1)	1.26688
5 C(13)	0.80460	23 C(13)	-0.61769	41 H(1)	-2.13757	59 H(1)	-2.78046
6 C(13)	0.80460	24 N(14)	18.10063	42 H(1)	-2.13757	60 H(1)	1.26688
7 N(14)	18.10085	25 C(13)	-16.77101	43 H(1)	-2.78054	61 H(1)	1.90847
8 C(13)	-16.77127	26 C(13)	-16.77102	44 H(1)	1.26690	62 H(1)	-3.30635
9 C(13)	-16.77128	27 C(13)	8.93835	45 H(1)	1.90851	63 H(1)	-0.06781
10 C(13)	8.93840	28 C(13)	-4.65712	46 H(1)	-3.30648	64 H(1)	1.71795
11 C(13)	-4.65737	29 C(13)	6.09124	47 H(1)	-2.78055	65 H(1)	1.30567
12 C(13)	6.09145	30 C(13)	-6.14693	48 H(1)	1.26690	66 H(1)	-0.06781
13 C(13)	-6.14719	31 C(13)	8.93835	49 H(1)	1.90851	67 H(1)	1.71797
14 C(13)	10.11652	32 C(13)	-4.65712	50 H(1)	-3.30648	68 H(1)	1.30566
15 C(13)	8.93841	33 C(13)	6.09122	51 H(1)	-0.06786	69 C(13)	10.11641
16 C(13)	-4.65738	34 C(13)	-6.14692	52 H(1)	1.30581	70 H(1)	1.90847
17 C(13)	6.09146	35 C(13)	10.11642	53 H(1)	1.71788	71 H(1)	-3.30634
18 C(13)	-6.14719	36 O(17)	-2.72127	54 H(1)	-0.06785	72 H(1)	-2.13757

CASSCF(3,3)/def2-TZVP results: Loewdin spin densities

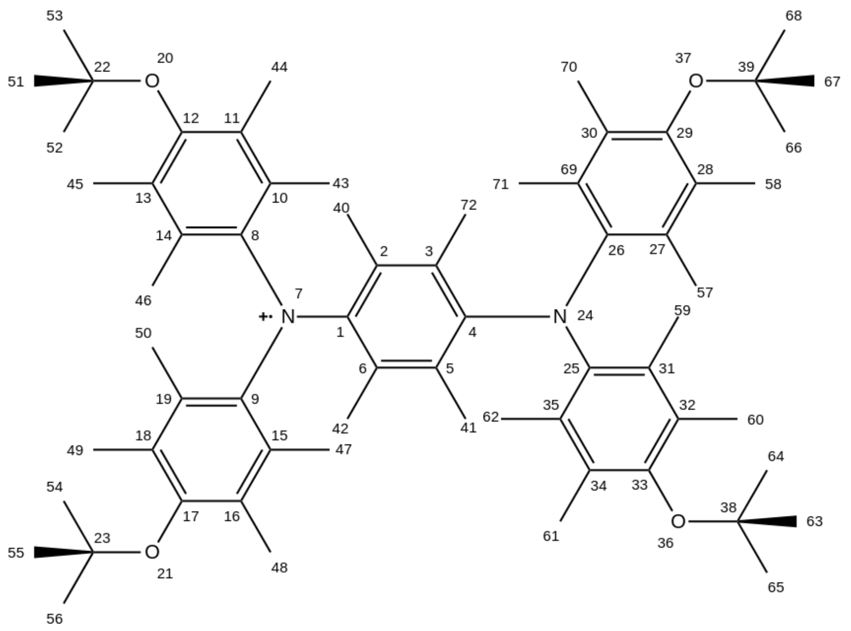

1 C 0.081212	19 C 0.035023	37 O 0.000539	55 H 0.000245
2 C 0.034608	20 O 0.007350	38 C 0.000066	56 H 0.000268
3 C 0.010948	21 O 0.007196	39 C 0.000079	57 H 0.000005
4 C 0.029455	22 C 0.001105	40 H 0.000651	58 H 0.000001
5 C 0.014506	23 C 0.001082	41 H 0.000339	59 H 0.000023
6 C 0.035018	24 N 0.015281	42 H 0.000650	60 H 0.000031
7 N 0.365500	25 C 0.003234	43 H 0.000661	61 H 0.000040
8 C 0.068956	26 C 0.001726	44 H 0.000198	62 H 0.000132
9 C 0.068516	27 C 0.000326	45 H 0.000211	63 H 0.000000
10 C 0.032065	28 C 0.000220	46 H 0.000819	64 H 0.000018
11 C 0.011452	29 C 0.003042	47 H 0.000640	65 H 0.000017
12 C 0.026451	30 C 0.003173	48 H 0.000199	66 H 0.000000
13 C 0.011756	31 C 0.001548	49 H 0.000211	67 H 0.000019
14 C 0.035657	32 C 0.001498	50 H 0.000801	68 H 0.000018
15 C 0.031541	33 C 0.001696	51 H 0.000000	69 C 0.001430
16 C 0.011302	34 C 0.001624	52 H 0.000248	70 H 0.000060
17 C 0.025903	35 C -0.001168	53 H 0.000275	71 H 0.000172
18 C 0.011471	36 O 0.000392	54 H 0.000001	72 H 0.000266

4. EPR results

The g-tensors of all studied radicals were obtained from simulation of G-band EPR spectra with EasySpin program[2] using function pepper, corresponding to the solid-state regime. Obtained g-tensor are listed in table.

**Radical**	**Solvent**	**Line width at 10% intensity**	**g tensor**
**BDPA**	TCE	70 MHz	2.00292<br>2.00269<br>2.00238

**1-4-amine**	TCE	130 MHz	2.00359<br>2.00309<br>2.00288
**1-3-amine**	TCE	115 MHz	2.00349<br>2.00296<br>2.00283

Table S1. Simulated g-tensors for the set of studied radicals.

Spectroscopic properties of studied radicals at 295 K in TCE: the isotropic hyperfine constants (a_{iso}, n - number of equivalent nuclei) and peak-to-peak line widths (LW). The X-band EPR spectra were simulated with EasySpin program[2] using function garlic.

**Radical**	**g_{iso}**	**a_{iso} / MHz**		**LW / mT**	
		^{1}H (n)	^{14}N (n)	Gaussian	Lorentzian
BDPA	2.002663	5.20 (4) 5.57 (4)	-	0.1102	0.1138
1-4-amine	2.003186		16.00 (2)	0.3480	0.1024
1-3-amine	2.003093	4.78 (2)	9.09 (2)	0.2106	0.2325

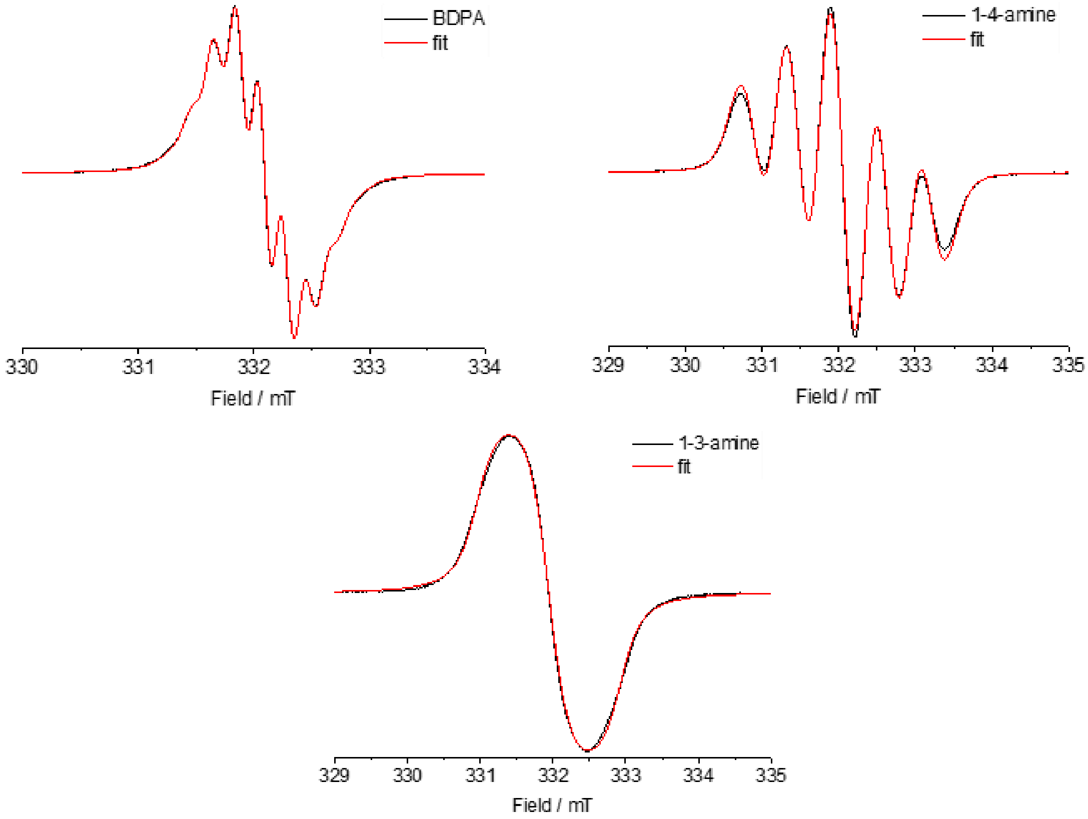

Figure S1. CW EPR spectra of radicals in TCE at room temperature.

5. DNP results

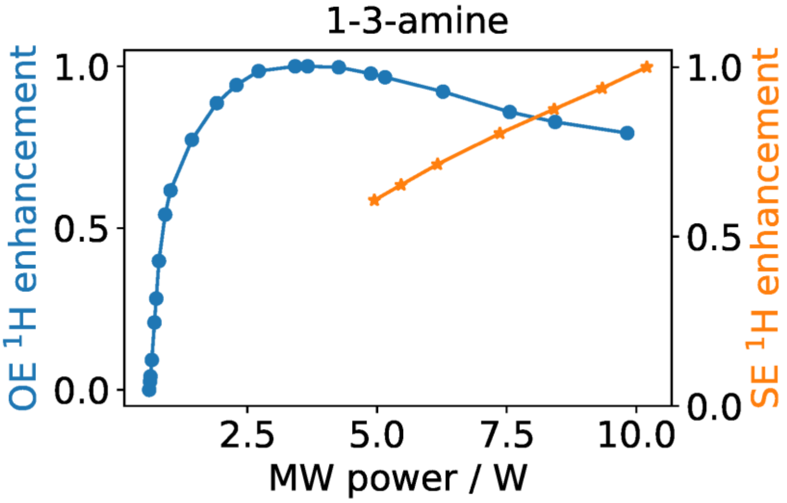

Figure S2. Enhancement as a function of MW power for different components of the field profile for 1-3-amine radical.

6. Literature

(1) Surry, D. S.; Buchwald, S. L. *J. Am. Chem. Soc.* **2007**, *129* (34), 10354 – 10355

(2) Stoll, S.; Schweiger, A. *J. Magn. Res*. **2006**, *178*, 42 – 55
